# Masculinity and men's health service use across four social generations: Findings from Australia's *Ten to Men* study

**DOI:** 10.1016/j.ssmph.2021.100838

**Published:** 2021-06-10

**Authors:** Jacquie McGraw, Katherine M. White, Rebekah Russell-Bennett

**Affiliations:** a School of Advertising, Marketing and Public Relations, Queensland University of Technology, Business School, B and Z Blocks, Gardens Point Campus, 2 George Street, Brisbane, QLD, 4000, Australia; b School of Psychology and Counselling, Faculty of Health, Queensland University of Technology, Brisbane, Australia; c School of Advertising, Marketing and Public Relations, Behavioural Economics, Society and Technology (BEST) Centre, Queensland University of Technology, Brisbane, Australia

**Keywords:** Men's health, Masculinity, Masculine norms, Health services, Social generations, Australia

## Abstract

There is a perception that traditional masculine ideals, usually thought deleterious for men's health outcomes, are no longer as relevant for younger social generations such as Millennials as they are for older social generations such as Baby Boomers. Yet, in Australia, there remains a disparity between younger men's and women's health outcomes and use of health services. Conformity to traditional masculinity is often cited as a barrier to men's positive health behaviours but conceptualisation of the construct is contested. We analysed a selected secondary dataset (*n* = 14,917) of Australian males aged between 15 and 55 years from *Ten to Men: The Australian Longitudinal Study on Male Health.* We examined the role of conformity to traditional masculine norms in predicting likelihood of regular primary and preventative health services use for different social generations. Analyses included mediated regression and adjusted logistic regression. Conformity to ten of the eleven specific traditional masculine norms predicted likelihood of increased or decreased regular health service use depending on the generation and health service type. Specific traditional masculine norms play a complex role in men's use of distinct health service types for different generations of Australian males. Practitioners wishing to increase men's engagement with health services should consider gender-sensitive approaches that leverage specific masculine norms relevant to the age cohort to drive positive outcomes in men's health.

## Highlights

• Conformity to specific traditional masculine norms predicts regular health services use for Millennials and Generation X.
• Influence of specific masculine norms is contextual to social generation and health service type.
• Conformity to overall traditional masculinity provides little explanation for men's health services use.
• Generation Z and Millennials have higher conformity to traditional masculine norms than Generation X and Baby Boomers.

## 1 Introduction

When controlling for sex-specific care, compared to women the same age, younger Australian men aged between 15 and 45 years are not regularly accessing health services including primary healthcare such as visiting a general practitioner (GP) with a health concern, and preventative health services such as visiting a GP just for a check-up when not sick (Australian Institute of Health and Welfare, 2019b; 2019c). In the younger age-groups between 15 and 45 years, males are dying at a greater rate, usually from preventable causes (Australian Institute of Health and Welfare, 2019a, 2019c; World Health Organization, 2018a). Despite availability of often subsidised health services, males in Australia, the United Kingdom, and around the World have lower life expectancy than females (World Health Organization, 2018b). In the study *Ten to Men: The Australian Longitudinal Study on Male Health*, 61% of surveyed Australian males aged 18–55 years said they did not visit their GP for a general health check-up at least once a year (Schlichthorst et al., 2016).

Masculine ideals such as being tough and self-reliant are often cited as barriers to men's use of health services, including primary health and preventative health services (King, Shields, Shakespeare, Milner, & Kavanagh, 2019; Novak, Peak, Gast, & Arnell, 2019). Indeed, for men, health behaviours can be seen as enactments of masculine identity and rejecting positive health behaviours such as seeking help for a physical or mental health problem, or even using sunscreen when outside, contribute to a ‘manly’ masculine identity construct (Courtenay, 2000, 2011; Ricciardelli & Williams, 2011). The damaging implications of traditional masculine ideals on men's health and wellbeing is being highlighted in the context of the COVID-19 pandemic with calls for health messages targeting men's health behaviours (Smith, Griffith, et al., 2020; White, 2020). However, while adherence to traditional masculine ideals is considered mostly incongruent with positive health behaviours, reimaging of some traditionally masculine traits such as being fit and muscular or a good role model or father-figure indicate a reconfigured masculinity construct may have some positive implications for men's health (Lewington, Sebar, & Lee, 2018; Mahalik, Di Bianca, & Sepulveda, 2020; Oliffe et al., 2019). Some argue, however, these modern takes on traditional masculinity could still be harmful to men's wellbeing, promoting unrealistic body images, for instance (Lewington et al., 2018).

Hegemonic masculinity is the dominant social construct of gender for men that influences male behaviours, including health behaviours (Courtenay, 2000). Connell (1995) challenged unidimensional thinking on the masculinity concept, proposing it be conceptualised as a multidimensional social construct. However, despite its variances, most iterations of masculinity have been drawn from hegemonic masculinity ideology that subordinates women and other masculinities, endorsing traditional dominant masculine norms often through risky, and unhealthy behaviours (Connell, 1995). Traditional masculinity is considered the idealised masculinity, yet the most damaging masculinity for men's health (Courtenay, 2011). For younger men, high-risk-behaviours such as drinking alcohol at dangerous levels, using illicit drugs, and engaging in unprotected sex have been associated with endorsing traditional masculinity ideals such as risk-taking and promiscuity (Courtenay, 2000; Courtenay & McCreary, 2011; Ricciardelli & Williams, 2011). For some older men, when such risky behaviours are less relevant, avoiding help-seeking and accessing preventative health services such as cancer screening services has also been associated with strong endorsement of traditional masculine norms like being tough and self-reliant (McGraw, Russell-Bennett, & White, 2019; Springer & Mouzon, 2011).

There are mixed views on whether an overall construct of masculinity provides enough insight into the drivers of men's health behaviours and attitudes, particularly for men from diverse cohorts (Levant, Wimer, & Williams, 2011). The global construct of traditional masculinity and traditional masculine norm conformity is often used in health contexts to understand men's health behaviours (Novak et al., 2019; Springer & Mouzon, 2011; Wong, Ho, Wang, & Miller, 2017). In health services use, it is not clear if the global construct of traditional masculinity is still relevant to understanding Australian men's access or avoidance of health services such as those for primary and preventative health.

Recent international studies have examined whether specific masculine norms can predict health behaviours such as the use of healthcare resources and preventative self-care (Levant & Wimer, 2014; Salgado, Knowlton, & Johnson, 2019). In the Australian context, where many health services are subsidised through the Australian Medicare Benefits Scheme (Australian Government, 2020), it is unknown whether conformity to specific traditional masculine norms plays a role in men's health service use for different age cohorts of men such as social generations.

In marketing and social research fields, social generations are often used to understand consumers born in similar time periods, of similar age and life stage and who have the same cultural influences from a certain span of time (Chaney, Touzani, & Slimane, 2017; McCrindle & Wolfinger, 2009). Social generations provide the social context for different age cohorts of a particular culture including technological, political, and economic influences as well as key events and popular culture (McCrindle & Wolfinger, 2009). Shared significant public health events, such as a pandemic like COVID-19 or epidemic like HIV/AIDS can also be influential in shaping a social generation (Settersten Jr et al., 2020; Wong, 2019).

Societal and cultural influences contribute significantly to the development of one's identity including masculine identity (Feldman, 2011). Therefore, the role of masculinity for different social generations may be a useful lens to examine men's health beliefs and behaviours, particularly to compare conformity to traditional masculine norms of the younger generations of Millennials, born between 1980 and 1994, and Generation Z, born between 1995 and 2009, with older generations comprising Baby Boomers, born 1946 to 1964, and Generation X, born 1965 and 1979 (Australian Bureau of AustralianBureau of Statistics, 2021; McCrindle & Wolfinger, 2009). For younger generations, changes in social constructions of gender and what it means to be a ‘man’ or ‘woman’ or to identify as ‘non-binary’ could indicate a shift in dominant gender norms (Donnelly & Twenge, 2017).

There are indications the role masculinity plays in men's health beliefs and behaviours could be changing for younger generations of men when accessing health services (Mitchell, 2018; Oliffe et al., 2019). However, there is limited examination of the role of conformity to masculine norms and ideals for different social generations in Australia when men use health services.

This study aims to firstly compare conformity to traditional masculine norms by younger generations of Australian males to older generations; secondly, to determine whether conformity to overall traditional masculinity plays a role in how males from different social generations access health services; and, thirdly, to understand if conformity to specific traditional masculine norms for males from different social generations plays a role in their regular use of either primary health services or preventative health services.

## 2 Methods

### 2.1 Study background

This study examined the role of traditional masculine norms for Australian men across different social generations when using primary and preventative health services. Analyses were conducted using secondary data from the first wave of *Ten to Men: the Australian longitudinal study for male health* (*Ten to Men*). *Ten to Men* is an Australian Government commissioned longitudinal study that commenced data collection in 2013/14 (Currier et al., 2016). Data collected in the original study included health behaviours, demographics, health service use and social attitudes including masculine ideals, measured by the 22-item Conformity to Masculine Norms Inventory (CMNI-22) (Mahalik, Locke, Ludlow, Diemer, Scott, Gottfried, 2003; Pirkis, Currier, Carlin, Degenhardt, Dharmage, Giles-Corti, 2016).

### 2.2 Data collection

The sampling strategy of the *Ten to Men* study was a stratified, multi-stage, cluster random sample (Pirkis et al., 2016). Researchers approached over 104,800 households (door-to-door canvasing) in 622 randomly selected statistical areas, recruiting 45,510 males in the study's scope (aged 10–55 years) ( Bandara, Howell, Silbert, et al., 2019; Pirkis et al., 2016). Data for Wave 1 were collected through hardcopy questionnaires for the Young Men and Adults surveys in 2013/14 (Currier et al., 2015). The *Ten to Men* datasets have a total of 16,021 respondents for Wave 1, encompassing three age-based cohorts (Bandara, Howell, Silbert, et al., 2019). More details about the *Ten to Men* study cohort and methods are published elsewhere (Currier et al., 2016; Pirkis et al., 2016). The current study used the Young Men (aged 15–17 years) and Adults (aged 18–55 years) datasets (*n* = 14,917). The surveys had some replicated items which enabled the current study to perform generational groupings for analyses including items reflecting health service use (Pirkis, English, & Currier, 2019).

### 2.3 Measures

#### 2.3.1 Health service use

The original Young Men and the Adults surveys included items for health service use in the last 12 months. Common to both surveys was the item: “*Excluding any time spent in hospital, have you consulted any of these health professionals for your own health in the past* 12 months*? Family doctor/General Practitioner (GP*). (yes/no)”. This item was used in the current study as the outcome variable for the construct of primary health service use. A ‘yes’ response was considered ‘regular use’.

Questions relating to preventative health service use were included only in the original Adults survey (aged 18–55 years, *n* = 13,891). The item used to indicate preventative health service use was: “*How often do you see your family doctor just for a check-up? That is, not because you are sick or injured, but to check on your general health. (1* = *More than once a year; 2* = *Once a year; 3* = *Less frequently; 4* = *Never)”.* The item was dummy coded where responses of 1 (more than once a year) and 2 (once a year) were considered ‘regular use’, and responses of 3 (less frequently) and 4 (never) considered a ‘no’ response. The current study defines ‘regular’ use of a health service as at least once a year in accordance with previous measures of ‘regular health service use’ used in Australian epidemiological and statistical data collection and in other published analyses using the *Ten to Men* data sets (Australian Bureau of AustralianBureau of Statistics, 2017,; Australian Institute of Health and Welfare, 2018; Schlichthorst et al., 2016).

#### 2.3.2 Traditional masculine norm conformity

Overall conformity to traditional masculine norms and conformity to specific traditional masculine norms was measured using the CMNI-22 instrument, an abbreviated version of the 11-factor 96-item CMNI (CMNI-96) using the two highest loading items from the original factors (Hamilton & Mahalik, 2009; Mahalik et al., 2003; Pirkis, Spittal, Keogh, Mousaferiadis, & Currier, 2017). The CMNI, including the CMNI-22, has been widely used to measure traditional masculinity ideologies in Westernised societies (Mahalik, Walker, & Levi-Minzi, 2007; Thompson & Bennett, 2015) The CMNI is designed to explore men's conformity to various dominant masculine norms (Mahalik, Talmadge, Locke, & Scott, 2005; Pirkis, Spittal, Keogh, Mousaferiadis, & Currier, 2017). The CMNI-22 Total score is the total of the 11 subscales scores and is used in this study as an indicator of overall conformity to traditional masculine norms (Mahalik et al., 2005). Each subscale represents a traditional masculine norm: importance of emotional control (*Emotional control*), endorsement of risk-taking (*Risk-taking*), importance of social status (*Status*), importance of being dominant (*Dominance*), salience of ‘playboy’ status (*Playboy*), salience of power over women (*Power over women*), primacy of work/school (*Work/school*), importance of self-reliance (*Self-reliance*), endorsement of violence as a resolution (*Violence*), importance of winning (*Winning*), and salience of heterosexual presentation (*Heterosexual presentation*) (Mahalik et al., 2003, 2005). The derived total scores for the CMNI-22 were provided in the original *Ten to Men* dataset. The subscales have demonstrated excellent concurrent validity and correlated well with the original 96-item scale (Hamilton & Mahalik, 2009; Pirkis, Spittal, Keogh, Mousaferiadis, & Currier, 2017; Thompson & Bennett, 2015). Reliability of the CMNI-22 scale in the current study was slightly low (Cronbach's alpha *α* = 0.66), which is considered within the generally acceptable range (Owen et al., 2010, p. 125) and consistent with other research using the abbreviated scale (Hamilton & Mahalik, 2009; Morrison, 2012; Owen et al., 2010). Pearson's correlations for each of the 2-item subscales ranged from low scores of *r* = 0.28 for *Work/school* to higher scores of *r* = 0.75 for *Emotional control*. The maximum overall raw score on the CMNI 22-question instrument is 66 and the maximum score for each subscale, or masculine norm, is 6. The total scores were converted to transformed scores (T-scores) for the analyses as recommended by the scale authors (Mahalik et al., 2005). T-scores can range from 0 to 100 where 50 indicates average confomrity, 50.01 to 60 reflects moderate conformity, 60.01 and above reflects extreme conformity, scores of 49.99 to 40 reflect moderate non-conformity, and scores 39.99 and below reflect extreme non-conformity to traditional masculine norms (Mahalik et al., 2005).

#### 2.3.3 Social generations

The current study derived social generations from participants’ ages using parameters for Australian social generations from the Australian Bureau of Statistics (ABS) (2021), and McCrindle and Wolfinger (2009). Across societies, there are variations on the specific birth year range for membership of each generation. However, our decision to use the ABS parameters enables future comparisons with Australian population data. It was determined that the year 2013 was the timepoint for selecting social generation groupings. The datasets for the current study included four Australian social generations: Baby Boomers (*n* = 2894), Generation X (*n* = 6221), Millennials (*n* = 4469), and Generation Z; (*n* = 1333). Of the Generation Z participants, *n* = 1026 completed the Young Men survey and *n* = 307 completed the Adults survey.

#### 2.3.4 Participant health and social characteristics

For the logistic regression analyses, variables from both surveys assessing mental and physical health, and demographic characteristics were included to control for other potential influences on health service use. The control variables included currently smoking, body mass index (BMI), derived from self-reported weight and height, hazardous alcohol consumption (Babor, Higgins-Biddle, Saunders, & Monteiro, 2001), and experiences of depression in the last 12 months, a self-reported dichotomous variable (Bandara, Howell, & Daraganova, 2019). Hazardous alcohol consumption measured self-reported consumption at hazardous or harmful levels in the past 12 months and was derived from the Alcohol Use Disorder Identification Test (Babor et al., 2001; Bandara, Howell, Silbert, et al., 2019). Demographic characteristics included remoteness of the area in which a participant lives (Australian Statistical Geography Standard Remoteness Area) such as ‘major city’, ‘inner regional’, and ‘outer regional’. Socio-economic status was controlled for using the Socio-Economic Index for Areas (SEIFA) Index of relative socio-economic disadvantage percentiles (Australian Bureau of AustralianBureau of Statistics, 2016). A low percentile indicated an area of high proportion of relatively disadvantaged people and a high percentile indicated an area of high proportion of relatively advantaged people (Australian Bureau of AustralianBureau of Statistics, 2016). The variable was recoded where a score ranging between 1 and 49.99 percent was recoded as ‘higher disadvantage’ and a score ranging between 50 and 100 percent was recoded as ‘lower disadvantage’. Aboriginal and/or Torres Strait Islander origin was measured as a self-reported categorical variable, and country of birth was also a self-reported variable recoded into a dichotomous variable of ‘Australia’ or ‘other’ (Bandara, Howell, & Daraganova, 2019).

Most control variables were duplicated in the Young Men and Adults surveys. However, the measure for ‘smoker’ in the Young Men survey was dummy coded from a five-category variable into a yes/no equivalent to the dichotomous variable in the Adults survey. The Young Men survey asked the average number of cigarettes smoked per day during the past four weeks (Bandara, Howell, & Daraganova, 2019). The present study dummy coded ‘0 cigarettes’ as ‘no’ for smoker, and all other selected responses ranging from ‘less than one cigarette per day’ to ‘ten or more cigarettes per day’ were coded as ‘yes’ for smoker. To control for participants' physical health in the last 12 months which could influence health service use, we created a variable ‘experienced injury, illness, surgery, or assault in past 12 months’ from the dichotomous item in the Adult survey “*In the past* 12 months*, have you experienced any of the following events? Serious personal injury, illness or surgery. (yes/no)*” and from the equivalent variable in the Young Men survey “*In the past* 12 months*, have you experienced any of the following events? You suffered a serious illness, injury or an assault. (yes/no)”* (Bandara, Howell, & Daraganova, 2019).

### 2.4 Analysis

Statistical analyses were conducted using IBM SPSS Statistics and Mplus. Subscales of the CMNI-22 were also treated as continuous variables. Mean scores of subscales and total scores for each social generation and all generations combined were compared in a series of one-way ANOVA tests. Two mediated regression analyses tested the indirect effect of the total T-scores of the CMNI-22 instrument, operationalising overall conformity to traditional masculine norms, on the relationship between social generation group and (1) primary health service use, and (2) preventative health service use. Controlling for health and social demographic characteristics, logistic regression analyses examined the likelihood of specific CMNI-22 subscales predicting (1) regular primary health service use and (2) regular preventative health service use. Logistic regression analysis was conducted on the whole dataset, representing ‘all generations’ and separated datasets for each social generation to independently capture different masculinity dimensions for each cohort. Consistent with recent publications from the *Ten to Men* dataset, analyses were not weighted as the present study is examining relationships between masculine norms and health service use rather than prevalence of a disease or risk factor in the population (Herreen, Rice, Currier, Schlichthorst, & Zajac, 2021; Milner, King, Scovelle, Currier, & Spittal, 2018; Spittal et al., 2016).

## 3 Results

### 3.1 Participant characteristics

Participant health and social characteristics for the whole sample and each social generation are presented in Table 1. Significance tests for independence between generations were also conducted. Across all generations represented, most participants had reported primary health service use by visiting a GP in the past 12 months (71%–85.7%). However, except for Baby Boomers, all generations had low regular preventative health service use through visits to a GP just for a check-up when not sick.

**Table 1:** Participant characteristics.

	All generations (*n* = 14,917)	Generation Z (*n* = 1333)	Millennials (*n* = 4469)	Generation X (*n* = 6221)	Baby Boomers (*n* = 2894)	Chi-square test of independence χ^2^
	Frequency (%)					
**Social generation membership:**						
Generation Z	1333 (8.9)					
Millennials	4469 (30.0)					
Generation X	6221 (41.7)					
Baby Boomers	2894 (19.4)					
**Age (years) mean**	36.59	16.45	26.27	41.21	51.87	
**Country of birth:**						
Australia	11479 (77.2)	116. (87.5)	3527 (79.2)	4616 (74.4)	2173 (75.4)	122.35^a^, ^d^
Other	3388 (22.8)	166 (12.5)	928 (20.8)	1586 (25.6)	708 (24.6)	
**Aboriginal and/or Torres Strait Islander:**						
Aboriginal	327 (2.2)	50 (3.8)	131 (2.9)	110 (1.8)	36 (1.2)	41.70^a^^,^^d^
Torres Strait Islander	22 (0.1)	1 (0.1)	7 (0.2)	9 (0.1)	5 (0.2)	
Both	23 (0.2)	4 (0.3)	7 (0.2)	10 (0.2)	2 (0.1)	
None	14423 (97.5)	1260 (94.5)	4295 (96.1)	6048 (97.2)	2820 (97.4)	
**Remoteness:**						
Major city	8779 (58.9)	755 (56.6)	2743 (61.4)	3640 (58.5)	1641 (56.7)	20.40^a^^,^^e^
Inner regional	3303 (22.1)	310 (23.3)	969 (21.7)	1368 (22)	656 (22.7)	
Outer regional	2833 (19.0)	268 (20.1)	756 (16.9)	1212 (19.5)	597 (20.6)	
**Socio-economic status:**						
Lower disadvantage	8072 (54.1)	741 (55.6)	2197 (49.2)	3479 (55.9)	1655 (57.2)	64.41^a^^,^^f^
Higher disadvantage	6843 (45.9)	592 (44.4)	2271 (50.8)	2741 (44.1)	1239 (42.8)	
**Smoker:**						
Yes	2829 (19.3)	92 (7.0)	936 (21.5)	1223 (19.9)	578 (20.2)	142.64^a^^,^^g^
No	11834 (80.7)	1215 (93.0)	3420 (78.5)	4922 (80.1)	2277 (78.7)	
**BMI mean and standard deviation (SD)**	27.40 (11.34)	23.03 (5.34)	26.50 (12.21)	28.28 (12.60)	28.59 (7.89)	82.84^a^^,^^j^^,^^b^
**Alcohol consumption:**						
Hazardous use	4902 (33.2)	214 (16.3)	1708 (38.9)	2105 (34.1)	875 (30.5)	244.80^a^^,^^h^
Not hazardous use	9853 (66.8)	1100 (83.7)	2688 (61.1)	4067 (65.9)	1998 (69.5)	
**Depression:**						
Experienced in past 12 months	1860 (12.8)	88 (7.0)	497 (11.3)	836 (13.7)	439 (15.7)	71.82^a^^,^^d^
Not experienced in the past 12 months	12684 (87.2)	1170 (93.0)	3888 (88.7)	5267 (86.3)	2359 (84.3)	
**Experienced injury, illness, surgery, or assault in past 12 months:**						
Yes	2116 (14.7)	152 (11.7)	603 (14.1)	893 (14.8)	471 (16.8)	20.282^a^^,^^i^
No	12301 (85.3)	1149 (88.3)	3670 (85.9)	2337 (85.2)	2337 (83.2)	
**Primary health service useVisit GP in the past 12 months:**						
Yes (regular use)	11594 (80.3)	923 (71.0)	3368 (75.4)	5183 (83.3)	2480 (85.7)	228.42^a^^,^^d^
No	2925 (19.7)	377 (29.0)	1098 (24.6)	1036 (16.7)	414 (14.3)	
**Preventative health service useVisit GP for check-up only in past 12 months**^c^**:**						
Yes (regular use)	5219 (39.2)	94 (32.9)	1195 (28.2)	2335 (38.9)	1595 (57.0)	589.74^a^^,^^h^
No	8103 (60.8)	192 (67.1)	3036 (71.8)	3671 (61.1)	1204 (43.0)	
**Overall conformity to traditional masculinity total score mean and standard deviation (SD)** **(score is out of 66)**	27.49 (SD = 5.69)	29.21 (SD = 5.97)	28.39 (SD = 5.84)	27.04.(SD = 5.43)	26.30 (SD = 5.49)	134.17^a^^,^^d^^,^^b^

a *P*-value < 0.001.

b F statistic.

c Only asked in Adults survey (n = 13,891).

d Significant differences between all generations.

e Significant differences between Millennials and Baby Boomers.

f Significant differences between Millennials, Generation X, and Baby Boomers.

g Significant differences between Generation Z and Millennials.

h Significant differences between Generations Z, Millennials, and Baby Boomers.

i Significant differences between Generation Z and Baby Boomers.

j Significant differences between all generations except between Generation X and Baby Boomers.

### 3.2 Generational conformity to traditional masculine norms

To address the first aim of the study, to compare conformity to traditional masculine norms by younger generations of Australian males to older generations, ANOVA tests were conducted comparing mean scores for each CMNI-22 masculinity subscale, total scores, and total T-scores between each generation (Table 2). Significance tests for independence between generations and post hoc analyses were also conducted. For specific traditional masculine norms *Status*, *Heterosexual presentation*, *Risk-taking*, *Violence*, *Winning*, and *Playboy*, the differences in mean scores between older generations (Generation X and Baby Boomers) and younger generations (Generation Z and Millennials) were statistically significant. Generation Z and Millennials' total mean scores were higher than Generation X and Baby Boomers’ total mean scores, with total T-scores indicating moderate conformity to traditional masculine norms for younger generations and moderate non-conformity for older generations (Mahalik et al., 2005). It should be noted that most mean scores for each subscale were either reflecting moderate conformity (3–4) or non-conformity (under 3) to the masculine norm.

**Table 2:** Means and standard deviations for CMNI-22 masculinity subscales and total scores.

CMNI-22 Subscale a	Generation Z	Millennials	Generation X	Baby Boomers	All generations
	Mean (SD)	Mean (SD)	Mean (SD)	Mean (SD)	Mean (SD)
**Status**	3.53 (1.11)	3.51 (1.04)	3.27 (0.97)	3.03 (1.04)	3.32 (1.03) b,d
**Heterosexual presentation**	3.43 (1.75)	2.79 (1.60)	2.90 (1.54)	3.07 (1.58)	2.95 (1.59) bc
**Emotional control**	3.30 (1.45)	3.15 (1.42)	3.14 (1.33)	3.23 (1.30)	3.17 (1.36) b,e
**Risk-taking**	3.02 (1.32)	2.98 (1.24)	2.68 (1.13)	2.49 (1.11)	2.76 (1.20) b,d
**Work/school**	3.02 (1.29)	2.76 (1.27)	2.53 (1.17)	2.53 (1.14)	2.64 (1.22) b,f
**Violence**	2.87 (1.46)	2.66 (1.46)	2.32 (1.43)	2.13 (1.45)	2.43 (1.46) b,c
**Winning**	2.53 (1.27)	2.56 (1.16)	2.44 (1.03)	2.31 (1.00)	2.46 (1.09) b,d
**Self-reliance**	2.47 (1.22)	2.63 (1.20)	2.58 (1.10)	2.62 (1.11)	2.59 (1.14) b,g
**Dominance**	2.41 (1.16)	2.56 (1.12)	2.49 (1.07)	2.36 (1.09)	2.48 (1.10) b,h
**Playboy**	1.70 (1.45)	1.72 (1.44)	1.54 (1.30)	1.45 (1.29)	1.59 (1.36) b,d
**Power over women**	1.13 (1.06)	1.26 (1.05)	1.28 (1.00)	1.27 (1.01)	1.26 (1.02) b,g
**Total score**	29.21 (5.97)	28.39 (5.84)	27.04.(5.43)	26.30 (5.49)	27.49 (5.69) b,c
**Total T-scores**	53.03 (10.49)	51.58 (10.26)	49.21.(9.54)	47.91 (9.64)	50.00 (10.00) b,c

a Subscale scores are out of 6.

b *P*-value < 0.001 for significance of independence between generational groups.

c Significant differences between all generations.

d Significant differences between all generations except Generation Z and Millennials.

e Significant differences between all generations except Generation Z and Baby Boomers; and Millennials and Generation X, and Baby Boomers.

f Significant differences between all generations except Generation X and Baby Boomers.

g Significant differences between all generations except Millennials and Generation X, and Baby Boomers; and Generation X and Baby Boomers.

h Significant differences between all groups except Generation Z and Generation X, and Baby Boomers.

### 3.3 The role of overall traditional masculinity

To determine whether conformity to overall traditional masculinity plays a role in how males from different social generations access health services (objective 2), two mediated regression analyses examined the effect of overall conformity to traditional masculine norms on firstly, primary health service use and secondly, preventative health service use. Mediated regression analyses found overall conformity to traditional masculine norms did not significantly mediate the relationship between social generation and primary health service use (*β* = 0.004, 95% CI 0.00–0.008). Overall conformity to traditional masculine norms had a small but weak negative indirect effect (*β* = −0.007, 95% CI -0.01 to −0.004) on the relationship between social generations and preventative health service use.

### 3.4 The role of specific traditional masculine norms

The third study objective, to understand if conformity to specific traditional masculine norms for males from different social generations plays a role in their regular use of either primary health services or preventative health services, was addressed through a series of logistic regression analyses conducted for each generational group and all generations combined. When adjusted for health and social demographic characteristics, logistic regression analyses found that, for different social generations and all generations combined, conformity to a total of ten specific traditional masculine norms affected likelihood of regular health services use for both primary health service use (Table 3) and preventative health service use (Table 4). Percentage statistics provided in Table 3, Table 4 were derived from the adjusted odds ratio (AOR) coefficient and indicate for every one unit increase in conformity to the CMNI-22 subscale, there is the stated percentage increase or decrease in likelihood of regular health services use. Sample sizes for each cohort analysed are included noting that for analyses for preventative health service use (Table 4), Generation Z was a considerably smaller sample (n = 201) because only the Adults survey was applicable.

**Table 3:** Conformity to specific traditional masculine norms predicting likelihood of regular primary health service use for social generations.

Social generation	Age at 2013/14 data collection	Traditional masculine norms predicting (%) increased regular primary health service use	AOR (95% CI)	Traditional masculine norms predicting (%) decreased regular primary health service use	AOR (95% CI)
**All Generations (n** = **11754)**	15–55 years	Winning (5.7%)	1.06 (1.00–1.11)*	Emotional control (8.3%)	0.92 (0.88–0.95)***
				Work/school (9.7%)	0.90 (0.87–0.94)***
				Self-reliance (8.6%)	0.91 (0.87–0.96)***
				Violence (4.6%)	0.95 (0.92–0.99)*
				Power over women (5.7%)	0.94 (0.89–0.996)*
**Generation Z** **(n** = **901)**	15–18 years	–	–	–	–
**Millennials (n** = **3394)**	19–33 years	Status (10%)	1.10 (1.01–1.20)*	Self-reliance (11%)	0.89 (0.82–0.96)**
		Winning (9.7%)	1.10 (1.01–1.19)*	Emotional control (11%)	0.89 (0.83–0.95)***
**Generation X (n** = **5120)**	34–48 years	Playboy (9.3%)	1.09 (1.02–1.17)**	Work/school (13.4%)	0.87 (0.81–0.93)***
				Power over women (12.3%)	0.88 (0.80–0.96)**
				Self-reliance (8.4%)	0.92 (0.85–0.99)*
**Baby Boomers (n** = **2339)**	49–55 years	–	–	–	–

Adjusted logistic regression analysis; total sample = 14,917; controlled for remoteness, body mass index (BMI), socioeconomic disadvantage, hazardous alcohol consumption, current smoker, injury, illness, surgery, or assault in last 12 months, depression in last 12 months, Aboriginal and/or Torres Strait Islander origin, country of birth not Australia.

AOR Adjusted Odds Ratio.

CI Confidence Interval.

*P-value < 0.05.

** *P*-value < 0.01.

***P-value < 0.001.

**Table 4:** Conformity to specific traditional masculine norms predicting likelihood of regular preventative health service use for social generations.

Social generation	Age at 2013/14 data collection	Traditional masculine norms predicting (%) increased regular preventative health service use	AOR (95% CI)	Traditional masculine norms predicting (%) decreased regular preventative health service use	AOR (95% CI)
**All Generations (n** = **10900)**	18–55 years	Work/school (8%)	1.08 (1.05–1.12) ***	Emotional control (17.9%)	0.82 (0.80–0.85) ***
		Winning (5.5%)	1.06 (1.01–1.10) *	Self-reliance (8.6%)	0.91 (0.88–0.95) ***
		Heterosexual presentation (3.8%)	1.04 (1.01–1.07)**	Status (11.1%)	0.89 (0.85–0.93) ***
**Millennials (n** = **3351)**	19–33 years	Work (12.5%)	1.13 (1.05–1.20)***	Emotional control (16.1%)	0.84 (0.79–0.89)***
		Power over women (10.7%)	1.11 (1.02–1.21)*		
		Risk taking (8%)	1.08 (1.01–1.16)*		
**Generation X (n** = **5044)**	34–48 years	Work (10.8%)	1.11 (1.05–1.17)***	Emotional control (20.4%)	0.80 (0.76–0.84)***
		Winning (8.5%)	1.09 (1.02–1.16)*	Self-reliance (10.9%)	0.89 (0.84–0.95)***
		Heterosexual presentation (4.6%)	1.04 (1.00–1.09)*	Violence (8.1%)	0.92 (0.88–0.96)***
				Status (6.7%)	0.93 (0.87–0.997)*
**Baby Boomers (n** = **2304)**	49–55 years	–	–	Emotional control (21.3%)	0.79 (0.73–0.85)***
				Status (11.4%)	0.90 (0.81–0.97)**
				Self-reliance (11.9%)	0.88 (0.81–0.96)**

Adjusted logistic regression analysis; total sample = 14,917; controlled for remoteness, body mass index (BMI), socioeconomic disadvantage, hazardous alcohol consumption, current smoker, injury, illness, surgery, or assault in last 12 months, depression in last 12 months, Aboriginal and/or Torres Strait Islander origin, country of birth not Australia.

AOR Adjusted Odds Ratio.

CI Confidence Interval.

*P-value < 0.05.

** *P*-value < 0.01.

***P-value < 0.001.

Hosmer and Lemeshow test results for all models indicated all were a good fit for the data. However, the omnibus χ2 test for the Generation Z preventative health service use model was not significant, χ2 (df = 20, n = 201) = 28.69, p = 0.094, indicating the predictor variables did not improve the predictive quality of the model. Post hoc power analysis with Power = 0.9, revealed the effect size (w = 0.34) was reasonable. However, a slightly larger sample (*n* = 232) would be needed to reach significance (*p* < 0.05).

## 4 Discussion

There are three key findings from the present analyses using the *Ten to Men* data of Australian males presented in this study: First, the overall global measure of traditional masculinity provides little explanation for men's use of health services. Secondly, conformity to specific traditional masculine norms predicts regular health service use for Millennial and Generation X males. Finally, the predictive roles of some specific traditional masculine norms have both positive and negative influences on regular health service use, depending on the service context and generation.

To understand the role of masculinity in men's health behaviours, such as accessing health services, a multidimensional instead of unidimensional perspective on masculinity may be more useful to allow a more fine-grained analysis of the impact of masculine norms on health service engagement. Masculinity is no longer seen as a unidimensional construct (Connell, 1995), nor is it fixed or static (Courtenay, 2000). Men's constructions of varying masculinities through the endorsement of traditional masculine norms, are not uniform (Connell, 1995; Courtenay, 2000). Previous masculinity research contends that global measures of masculinity do not adequately reveal the varied nuances of the construct, particularly in health contexts (Mahalik et al., 2005; Owen, 2011; Wong et al., 2017). Wong et al.’s (2017) meta-analyses examining relationships between conformity to masculine norms and mental health outcomes found the generic construct of masculine norms, which they operationalised through various versions of the CMNI, was unhelpful in explaining outcomes compared to specific dimensions of masculinity. The findings of the present study demonstrate that, while overall conformity to traditional masculine norms is relevant for different generations of Australian males, as a measure of association for health services use, it offers negligible meaningful explanation. Examination of conformity to specific masculine norms for different generations' health service use, however, results in a richer depiction of likely influences.

This study found conformity to specific traditional masculine norms will predict different outcomes in health service use that are unique to social generation and the type of service. Each generation had a different predictive model for both regular primary health service use and regular preventative health service use, and none of the generations' models were replicated with the ‘all generations’ combined samples. As Table 3, Table 4 show, specific traditional masculine norms that were highly significant (*p* < 0.001) for predicting different generations' health service use included *Emotional control*, *Work/school*, *Self-reliance*, *Status*, and *Violence*. Previous studies using the CMNI instrument have also found the same specific masculine norms to be associated with men's health behaviours (Mahalik et al., 2007; Levant & Wimer, 2014; Salgado et al., 2019).

Additionally, this study found some specific traditional masculine norms predict increased likelihood of regular health service use. Millennial and Generation X models for primary and preventative health service use included seven specific traditional masculine norms positively associated with regular health services use. Traditional masculinity is mostly associated with negative health behaviours (Addis & Mahalik, 2003; Courtenay, 2000, 2011; Springer & Mouzon, 2011). However, emerging studies have explored the potential for the endorsement of some masculine norms to predict positive health behaviours such as increased use of mental health services and preventative self-care (Levant et al., 2011; Salgado et al., 2019; Sileo & Kershaw, 2020). When exploring whether theoretical positive masculinity constructs are more broadly socially accepted and enacted, McDermott et al. (2019) found some traditional masculine ideals usually captured as negative traits can to a degree be associated with socially acceptable responses or positive behaviours, such as being successful in one's job. The present study adds to this avenue of men's health research from the perspective of primary and preventative health service use, suggesting conformity to some traditional masculine norms could lead to regular use of certain health services. For instance, findings suggest Generation X males with higher conformity to traditional norms *Work/school* (AOR 1.11, 95% CI 1.05–1.17) and *Winning* (AOR 1.09, 95% CI 1.02–1.16), masculine norms associated with competitiveness and achievement, are likely to keep their health in check by regularly accessing preventative health services such as going to the GP for a check-up. This finding suggests a link between traditional masculine ideals of male competitiveness with positive preventative health behaviours. Increased conformity to *Work/school* (AOR 1.13, 95% CI 1.05–1.20) was also likely to predict regular preventative health service use for Millennial males, as was *Power over women* (AOR 1.11, 95% CI 1.02–1.21). In a report comparing important issues for Australia's Millennials (referred to as Generation Y) and Generation X cohorts, Chester et al. (2018) found that next to the environment, job security was the most important issue for the younger generation, more so for the males in the cohort. Generation Y participants reported job insecurity and housing affordability threatened to inhibit their progress through life stages such as marriage and starting a family (Chester, 2018). One Generation Y male expressed concerns on fulfilling expectations to be the sole provider for his partner and family (Chester, 2018). Some U.S. social researchers suggest there is a ‘stalling’ of the gender revolution since the mid-1990s, with a return to more conventional attitudes to women in the workforce from younger generations, particularly among young men (Fate-Dixon, 2017; Pepin & Cotter, 2018). This phenomenon might explain the findings in the current study, where Australian Millennial males with higher conformity to *Work/school* and *Power over women* are more likely to regularly use a preventative health service, possibly to ensure they remain fit for work and fulfil ‘breadwinner’ expectations in an uncertain job market (Coontz, 2017; Fate-Dixon, 2017).

Of note in this study is some specific traditional masculine norms change directional roles in predicting health service use for different generations. For instance, conformity to specific norm *Work/school* changes from a positive to negative influence depending on the health service context. This finding suggests that, for some males accessing a primary health service when one might be sick or injured has very different implications to one's masculine identity than when accessing a preventative health service for a check-up when one is not sick. Men with traditional masculine ideals have been found to overestimate their health status and downplay illness or injury, avoiding accessing care even when sick (Courtenay, 2011; Leone, Rovito, Mullin, Mohammed, & Lee, 2017; Springer & Mouzon, 2011). Additionally, higher conformity to *Power over women* (AOR 1.11, 95% CI 1.02–1.21) predicted increased likelihood of preventative health service use for Millennial males but reduced regular primary health service use for Generation X males (AOR 0.88, 95% CI 0.80–0.96). The already suggested association with ‘breadwinner’ expectations might explain this positive influence for Millennial men (Fate-Dixon, 2017), whereas Generation X males who endorse traditional masculine ideals of male dominance and strength over women, including in the workplace, may be more likely to avoid going to the doctor even when sick or injured to avoid appearing weak or not fit for work (Courtenay, 2011). While conformity to *Power over women* is a problematic norm for men's health (Courtenay, 2000, 2011), and gender relations (Jewkes, Flood, & Lang, 2015), its significance in this research serves to highlight polarised shifts in attitudes occurring within younger generations of males (Fate-Dixon, 2017; Pepin & Cotter, 2018). The duality of some specific norms for either positive or negative influences on health services use highlights the complexity of each dimension of masculinity. In their replicated study of Levant et al. (2011), investigating associations between specific traditional masculine norms and health behaviours, Levant and Wimer (2014) found that some aspects of masculinity can be positively associated with certain health behaviours, and negatively associated with others. However, the associations are highly contextual to the behaviour, the sample, and the particular masculine trait (Levant and Wimer, 2014). The present study also finds that associations between conformity to some dimensions of masculinity and health services use are sensitive to service type and generation.

The current study provides practitioners wishing to engage males from different age cohorts with some key insights into the workings of the varied dimensions of traditional masculinity when men decide on health service use. Findings could inform tailored, gendered approaches to influence men's use of services fundamental to improved health outcomes. However, findings also serve to caution practitioners when targeting males that, depending on the health service or behaviour promoted, specific traditional masculine norms can serve either as a motivator or barrier to positive health behaviours for some generations and health service contexts. While some theorists warn health practitioners of the risks that come with messaging that endorses traditional masculine norms (Fleming, Lee, & Dworkin, 2014), there are also proponents for a gendered approach to men's health (Courtenay, 2011; Oliffe et al., 2019; Smith, Watkins, & Griffith, 2020; Wong et al., 2017). This study supports scrutinised and focussed gender-sensitive approaches that explore the potential to reconfigure some specific masculine norms to drive positive outcomes in men's health. For instance, public health messaging targeting Millennial males could associate regular preventative skin cancer screening with workplace comradery.

### 4.1 Strengths and limitations

The use of the *Ten to Men* data enabled the examination of a large sample size with an extensive household recruitment process, a broad age range that encompasses four social generations, and the availability of key health and socioeconomic control variables (Bandara, Howell, & Daraganova, 2019; Milner et al., 2018). The present study has limitations, however, to consider when assessing the findings. The CMNI-22 instrument had slightly low reliability for its global scale and had some low reliability scores with some subscales. A longer version like the CMNI-46 may have increased reliability. In public health contexts, however, and in particular given the large scope of the original *Ten to Men* research project, the abbreviated instrument was more efficient (McDermott et al., 2019). The CMNI-22 uses the two highest loading items for each subscale taken from the original CMNI-94 (Mahalik et al., 2003). The absence of a preventative health service use variable in the Young Men's survey reduced representation of Generation Z in the preventative health service use analyses to males aged 18 years. However, the *Ten to Men* Generation Z cohort was aged between 10 and 18 years at the data collection time point and accessing preventative health services may not have been as applicable for the younger males compared to adult males.

## 5 Conclusions

This study contributes to research and practice by revealing that in the context of health services use, specific dimensions of masculinity are more useful to understanding men's health behaviours than the global construct. However, specific traditional masculine norms influential in health services use differ for each social generation, and higher conformity to some specific norms predicts increased use of health services for some generations. The predictive role of some specific norms also changes from a positive influence to a negative influence in regular health service use depending on the generation and service type. Findings provide insights into the multidimensional role of specific traditional masculine norms for men's health service use, which potentially reflects differing attitudes of social generations. Such insights will be useful for practitioners in public health and other disciplines wishing to engage males, particularly Millennial and Generation X males.

Existing disparities in men's health outcomes, which have become more deleterious with the effects of the COVID-19 pandemic, prompt further calls to better engage men in protective health behaviours through preventative health messaging (Smith, Griffith, et al., 2020; White, 2020). It is crucial practitioners are mindful of the divergent drivers of men's health behaviours such as masculinity constructs, and the varied roles they play for different age cohorts, including their potential to be leveraged to improve health outcomes across the lifespan.

## Ethics approval statement

The *Ten to Men: the Australian longitudinal study for male health* study reported in this paper received ethics approval from the University of Melbourne Human Research Ethics Committee (HREC 1237897 and 1237376). Participants provided written consent for their participation. The Queensland University of Technology's University Human Research Ethics Committee has assessed the research for this reported research as meeting the conditions for exemption from HREC review and approval in accordance with section 5.1.22 of the National Statement on Ethical Conduct in Human Research (2007) (Exemption number: 1900000824).

## Consent for publication

Not applicable.

## Availability of data

The data (Ten to Men: the Australian longitudinal study for male health) that support the findings of this study are available from the Australian Institute of Family Studies via a request and review process. Information on data access along with Wave 1 surveys, data books, and Data User's Manual are available at http://www.tentomen.org.au/index.php/researchers.html.

## Funding statement

JM was supported by an 10.13039/100015986Australian Government Research Training Program (RTP) Scholarship.

## Author's contribution statement

JM, KW, and RRB were responsible for the analytical design. JM undertook data analysis. JM, KW, and RRB were involved with analysis interpretation. JM drafted the manuscript. All Authors undertook revision of the manuscript and approved the submitted manuscript.

## Declaration of competing interest

The authors declare no conflicts of interest or competing interests in connection with this article.
